# Puerarin ameliorates metabolic dysfunction-associated fatty liver disease by inhibiting ferroptosis and inflammation

**DOI:** 10.1186/s12944-023-01969-y

**Published:** 2023-11-24

**Authors:** Mengmeng Yang, Longqing Xia, Jia Song, Huiqing Hu, Nan Zang, Jingwen Yang, Ying Zou, Liming Wang, Xiaoyue Zheng, Qin He, Jidong Liu, Fuqiang Liu, Kai Liang, Lei Sun, Li Chen

**Affiliations:** 1 Department of Endocrinology, Qilu Hospital of Shandong University, Jinan, 250012 Shandong China; 2 Institute of Endocrine and Metabolic Diseases of Shandong University, Jinan, Shandong China; 3 Key Laboratory of Endocrine and Metabolic Diseases, Shandong Province Medicine & Health, Jinan, Shandong China; 4 Jinan Clinical Research Center for Endocrine and Metabolic Disease, Jinan, Shandong China

**Keywords:** Metabolic dysfunction-associated fatty Liver Disease, Puerarin, SIRT1, Nrf2, Ferroptosis, Inflammation

## Abstract

Metabolic dysfunction-associated fatty liver disease (MAFLD) is frequently linked to type 2 diabetes mellitus (T2DM), and both conditions exacerbate the progression of the other. However, there is currently no standardized treatment or drug for MAFLD. In this study, A MAFLD animal model through a high-fat diet (HFD) along with administration of streptozotocin (STZ), and palmitic acid (PA)-induced AML12 cells were treated by puerarin. The objective of this study was to assess the therapeutic effect of puerarin, a flavonoid substance that possesses various pharmacological properties, on MAFLD. The results showed that puerarin administration enhanced glucose tolerance and insulin sensitivity, while also mitigating liver dysfunction and hyperlipidemia in MAFLD mice. Moreover, puerarin attenuated oxidative stress levels and inflammation in the liver. Transmission electron microscopy and Western blot analysis indicated that puerarin inhibited ferroptosis in vivo. Further mechanistic investigations revealed that puerarin upregulated SIRT1 expression, increased nuclear factor erythroid 2-related factor 2 (Nrf2) protein levels, and facilitated translocation into the nucleus. The protective effect of puerarin on PA-induced AML12 cells was diminished by the utilization of EX-527 (a SIRT1 inhibitor) and Nrf2 siRNA. Overall, the results demonstrate that puerarin ameliorates MAFLD by suppressing ferroptosis and inflammation via the SIRT1/Nrf2 signaling pathway. The results emphasize the possible medicinal application of puerarin for managing MAFLD.

**Supplementary Information:**

The online version contains supplementary material available at 10.1186/s12944-023-01969-y.

## Introduction

The liver serves as the metabolic center for factors such as lipids, glucose, and amino acids. Imbalances in these factors can cause liver insulin resistance and liver diseases. Liver diseases are major global causes of mortality [1], particularly metabolic dysfunction-associated fatty liver disease (MAFLD). The increasing global incidence of MAFLD has resulted in significant medical and economic burdens. However, the lack of standardized treatment or effective medications underscores the need for in-depth exploration of MAFLD pathogenesis to identify new therapeutic targets and develop effective MAFLD treatments.

MAFLD is closely linked to various chronic metabolic diseases, including type 2 diabetes mellitus (T2DM). MAFLD and T2DM share similar pathogenesis, both of which are the result of metabolic syndrome, and the development of both diseases is linked to insulin resistance [2]. There is substantial evidence supporting the mutual influence of MAFLD and T2DM. The progression of MAFLD to hepatic fibrosis and hepatocellular carcinoma is strongly linked to T2DM [3], and individuals with T2DM are at a higher risk of all-cause mortality and cardiovascular disease due to the presence of MAFLD [4]. In addition, there is a reduced survival rate in patients with chronic liver disease due to the presence of extrahepatic cancers [5]. Therefore, it is imperative to identify the underlying causes and comprehend the development of MAFLD in order to devise interventions and treatments for both MAFLD and associated metabolic disorders.

Ferroptosis, a form of programmed cell death discovered recently, plays a major role in MAFLD progression [6, 7]. The liver is responsible for storing approximately 20–25% of total iron storage in the body, making it an essential organ for iron storage and maintaining iron homeostasis [8]. Iron overload and lipid peroxidation are the two primary mechanisms that contribute to ferroptosis. Polyunsaturated fatty acids (PUFAs) play a crucial role in the structure of cell membranes. They can bind with coenzyme A to form PUFA-CoA and further participate in esterification to produce the membrane phospholipid of polyunsaturated fatty acids (PUFA-PL). PUFA-PL is susceptible to peroxidation to produce harmful lipid peroxides that cause irreversible damage to cell membranes, resulting in cellular ferroptosis [9]. The Fe^3+^ can bind to the transferrin and enter the cytoplasm after being recognized by the transferrin receptor. Some iron can be stored in ferritin. When iron homeostasis is out of balance, ferritin is transported to lysosomes for ferritinophagy, and then free iron ions are released into cytoplasm [10]. Fe^2+^ is extremely unstable and easily oxidized. Fe^2+^ can participate in Fenton reaction will further cause the peroxidation of PUFA-PL and lead to ferroptosis [11]. Hepatic insulin resistance and MAFLD can elevate iron levels in hepatocytes [12]. Iron can participate in intracellular redox reactions to produce oxygen radicals such as reactive oxygen species (ROS) [13]. Moreover, the susceptibility of PUFAs in cell membranes and organelles to ROS attack and subsequent lipid peroxidation can trigger ferroptosis in cells [14]. Different lipid peroxidation products can contribute to the progression of MAFLD; for example, malondialdehyde (MDA) and 4-hydroxynonenal (4-HNE) are involved in different stages of MAFLD. There is compelling evidence indicating that MDA levels in the bloodstream are higher in MAFLD patients compared to controls [15], and HNE significantly correlates with liver fibrosis [16]. In MAFLD, inflammation and fibrosis are two key pathophysiological processes. The presence of iron enhances the respiratory burst activity of Kupffer cells, potentially leading to a proinflammatory impact by activating nuclear factor (NF)-κB. Consequently, this activation induces hepatic synthesis of various proinflammatory and fibrotic mediators [17]. Research has shown that hepatic ferroptosis plays a vital part in initiating inflammation related to MAFLD and inhibiting ferroptosis can effectively prevent liver fibrosis induced by a high-fat diet (HFD) or CCl_4_ [18]. These findings suggest that ferroptosis is an additional therapeutic target for MAFLD. Inhibition of ferroptosis may serve as a viable therapeutic strategy for managing MAFLD.

SIRT1, which is a type of NAD-dependent deacetylase, is involved in energy regulation, mitochondrial biosynthesis, inflammation regulation, and oxidative stress [19, 20]. The transcription factor nuclear factor erythroid 2-related factor 2 (Nrf2) regulates oxidative stress and works together with its downstream target gene HO-1 to preserve the balance of redox within cells. Interestingly, Nrf2 has been shown to be a negative regulator of ferroptosis [21]. SIRT1 has been shown to deacetylate Nrf2 and regulate the activity of Nrf2 to inhibit oxidative stress. However, the role of SIRT1 in regulating cellular ferroptosis through Nrf2 remains relatively understudied.

Traditional Chinese medicine offers several advantages in treating chronic metabolic diseases, including its natural acquisition, affordability, and fewer side effects. Puerarin, which is a type of flavonoid, possesses several pharmacological characteristics, including cardioprotection, reducing insulin resistance, and inhibiting inflammation and oxidative stress [22–24]. Studies have shown that puerarin can treat a wide range of chronic diseases, including diabetes [24], colitis [25], and NAFLD [26]. Although study has confirmed that puerarin improves MAFLD by reducing lipid deposition, however, it did not observe whether puerarin had antiferroptotic and anti-inflammatory effect on MAFLD [26]. Therefore, we hypothesized that puerarin may ameliorate MAFLD by inhibiting ferroptosis and inflammation. Additionally, the involvement of SIRT1 is crucial in mediating the beneficial impacts of puerarin.

During this study, a MAFLD mouse model was established by subjecting mice to a HFD combined with streptozotocin (STZ) injection. AML12 cells were stimulated with palmitic acid (PA) to create a cellular MAFLD model in vitro, with the aim of exploring the effect of puerarin on ferroptosis in MAFLD. According to results, puerarin improved MAFLD by regulating the SIRT1/Nrf2 signaling pathway to inhibit ferroptosis and inflammation, thus highlighting the potential of puerarin as a treatment for MAFLD and presenting new scientific data for future clinical approaches to MAFLD treatment.

## Methods

### Animals

The Model Animal Research Center of Shandong University (Jinan, China) supplied forty male C57BL/6 J mice aged four weeks for this study. The mice were fed in a standard environment at 22–25 °C with 55%±5% humidity and a cycle of light and dark lasting for 12 h. Following two weeks of normal chow diet feeding, the mice were equally divided into the normal chow diet (control) and HFD (Jiangsu Xietong Corporation, Nanjing, China, Table S1. Compositions of experimental diets) groups. Following 12 weeks of uninterrupted feeding, the HFD-fed mice were administered STZ by intraperitoneal injection to establish the mouse model of MAFLD. After fasting glucose ≥ 16.7 mmol/L was measured twice, modeling success was assessed. The control mice were separated into two groups: Control + Vehicle (n = 8) and Control + Puerarin (n = 8). The MAFLD mice were separated into two groups: MAFLD + Vehicle (n = 8) and MAFLD + Puerarin (n = 8). Puerarin (200 mg/kg BW, CAS No: 3681-99-0, MedChemExpress) was prepared in a vehicle (sodium carboxymethylcellulose) and administered by gavage. The treatment frequency was three times per week (Monday, Wednesday, and Friday). The Control + Vehicle group and MAFLD + Vehicle group were given an equal volume of vehicle without puerarin. Body weights were examined weekly, and after four weeks, the mice were subjected to the intraperitoneal glucose tolerance test (IPGTT) and intraperitoneal insulin tolerance test (IPITT), at the end of which the mice were anesthetized and sacrificed to collect blood and liver samples.

### Metabolic index detection

The mice were given a glucose injection intraperitoneally to perform the IPGTT after 12–16 h of fasting and were administered insulin to perform the IPITT following 4–6 h of fasting. The IPGTT and IPITT were performed by collecting blood from the tail vein tip at 0 min and 30, 60, 90, 120, 150, and 180 min after administration to determine the glucose level. ELISA kits (Nanjing, Jiancheng) were used to measure aspartate triglyceride (TG), total cholesterol (TC), aminotransferase (AST), and alanine aminotransferase (ALT) levels in the blood serum. Catalase (CAT) was measured by visible light method, malondialdehyde (MDA) was measured by thiobarbituric acid method, glutathione peroxidase (GSH-PX) was measured by colorimetric method, and total superoxide dismutase (T-SOD) was determined by hydroxylamine method in the homogenate of liver tissue using reagent kits (Nanjing, Jiancheng), complying with the manufacturer’s guidelines.

### Cell culture

The alpha mouse liver 12 (AML12) cell line was procured from Shanghai Cell Bank, cultured in DMEM/F12 medium (Gibco, USA) containing 10% fetal bovine serum, 100 U/mL penicillin, 100 μg/mL streptomycin, 1% insulin-transferrin-selenium, and 0.1 μmol/L dexamethasone, and then placed in an incubator at 37 °C with 5% CO_2_. The MAFLD cell model was established using 0.4 mM PA to stimulate AML12 cells for 48 h in the presence of puerarin. The cells were pretreated with EX-527 (a SIRT1 inhibitor, CAS. No. 49843-98-3, MedChemExpress) and Nrf2 siRNA. The siRNA sequences were provided by GenePharma. The Nrf2 siRNA sequences were as follows: sense 5′-GGAGGCAAGACAUAGAUCUTT-3′ and antisense 5′-AGAUCUAUGUCUUGCCUCCTT-3′. The negative control sequences were as follows: sense 5′-UUCUCCGAACGUGUCACGUTT-3′; antisense 5′-ACGUGACACGUUCGGAGAATT-3′. AML12 cells were transfected with siRNA by using Lipofectamine 3000 according to the manufacturer’s guidelines.

### Western blot analysis

Radioimmunoprecipitation assay (RIPA) lysis buffer was used to lyse AML12 cells and liver tissue. A BCA assay kit (Beyotime, China, CAS. No. P0012S) was used to determine protein concentrations, after which the proteins were separated on sodium dodecyl sulfate‒polyacrylamide gels and transferred to polyvinylidene difluoride (PVDF) membranes (R1PB86935, 0.22 μm, Millipore, USA). The PVDF membranes were incubated with 5% skim milk for 1 h at room temperature, after which the membranes were incubated with specific primary antibodies at 4 °C overnight. The following day, horseradish peroxidase-conjugated secondary antibodies were added and incubated with the membranes for 1 h at room temperature. Enhanced chemiluminescence was used to display the protein bands and quantify them using ImageJ software, and β-actin was used as a standard. The specific primary antibodies were as follows: SIRT1 (1:1000, Abcam, USA, Cat. No. ab189494), p-Nrf2 (Ser 40) (1:1000, Affinity, China, Cat. No. DF7519), Nrf2 (1:1000, CST, USA, Cat. No. 12,721), HO-1 (1:1000, ABclonal, China, Cat. No. A1346), SLC7A11 (1:1000, ABclonal, China, Cat. No. A2413), GPX4 (1:1000, Abcam, USA, Cat. No. ab125066), phospho-NF-κB p65 (1:1000, CST, USA, Cat. No. 3033T), NF-κB p65 (1:1000, CST, USA, Cat. No. 8242T), TNF-α (1:1000, Bioss, China, Cat. No. bs-2081R), IL-1β (1:100, Abcam, USA, Cat. No. ab283818), IL-6 (1:100, Abcam, USA, Cat. No. ab290735), and β-actin (1:1000, Abways, China, Cat. No. Ab0035).

### Coimmunoprecipitation assay

AML12 cells were seeded in 6 cm Petri dishes, and the cells were harvested and lysed on ice with immunoprecipitation lysis buffer, followed by centrifugation at 4 °C for 10 min at 13,000 g. The supernatant was collected and incubated with rabbit IgG or primary antibodies overnight at 4 °C. The following day, preequilibrated magnetic beads were added and incubated at room temperature for 1 h. Then, the beads were rinsed four times with lysis buffer, and finally, the immunoprecipitants were resuspended in 1X SDS loading buffer and boiled at 95 °C for 10 min. The beads were detached, and the supernatant was collected for western blotting.

### Assessment of liver pathology

After the mice were anesthetized, liver tissues were harvested and stored at -80 °C or fixed in a 4% paraformaldehyde solution for subsequent staining. Following fixation in paraformaldehyde, liver tissues were further embedded in paraffin and sliced into 5-μm-thick slices. After typical dewaxing by standard techniques, hematoxylin-eosin (HE) was used to stain the paraffin sections to assess histology and vacuolar and fatty changes in the liver. Masson and Sirius red staining was used to assess fibrosis in the liver.

### Immunostaining

For immunofluorescence staining, the liver paraffin sections were dewaxed, and antigen retrieval was performed using citrate buffer, followed by antigenic closure at room temperature with 10% goat serum. After 1 h of closure, the liver paraffin sections were incubated with p-Nrf2 antibodies (1:100, Affinity, China, Cat. No. DF7519) overnight at 4 °C. Subsequently, a fluorescent secondary antibody was added and incubated at room temperature for 60 min, followed by 5 min of DAPI staining, and the paraffin sections were observed under a fluorescence microscope (Olympus, Japan, BX53).

For immunohistochemical staining, the liver paraffin sections were dewaxed, and citrate buffer was used for antigen retrieval. Peroxidase activity was inhibited with a 3% hydrogen peroxide solution. Immediately after antigen closure with 10% goat serum at room temperature, the liver paraffin sections were incubated for 1 h, followed by overnight incubation at 4 °C with SIRT1 (1:100, Affinity, China, Cat No. DF7519), F4/80 (1:100, Abcam, USA, Cat. No. ab3004216), IL-1β (1:100, Abcam, USA, Cat. No. ab283818), and IL-6 (1:100, Abcam, USA, Cat. No. ab290735) antibodies. Subsequently, the liver paraffin sections were incubated with secondary antibodies at room temperature for 1 h, followed by DAB staining and hematoxylin staining. Finally, the staining was observed under a microscope.

### Transmission electron microscopy (TEM)

As previously described, liver tissue from mice was examined by TEM [27], and the mitochondrial morphology of hepatocytes was observed.

### Real-time quantitative PCR analysis

TRIzol reagent was used to obtain RNA from liver and AML12 cells, and a spectrophotometer was used to determine RNA concentrations and purity. Reverse transcription was performed using the Prime Script RT Reagent Kit (Takara, Japan, Cat. No. RR047A) according to the manufacturer’s protocols. RT‒qPCR was performed with the SYBR Green PCR Kit, and changes in gene expression were compared to the control and calculated using Eq. 2^− ρρCT^. Uniform labeling was performed using *β-actin*, and the primer sequences were as follows: *Tnf-α*, sense 5′- ATCTTCTCAAAATTCGAGTGACAAC-3′ and antisense 5′- TGGGAGTAGACAAGGTACAACCC-3′; *Il-1β*, sense 5′- AGGCCACAGGTATTTTGT-3′ and antisense 5′- GCCCATCCTCTGTGACTC - 3′; *Il-6* sense 5′- GCTACCAAACTGGATATAATCAGGA - 3′ and antisense 5′- CCAGGTAGCTATGGTACTCCAGAA - 3′; and *β-actin*, sense 5′- AGCCATGTACGTAGCCATCCA - 3′ and antisense 5′- TCTCCGGAGTCCATCACAATG - 3′.

### Molecular docking analysis

The Yinfo Cloud Computing Platform (https://cloud.yinfotek.com/) was used to perform docking calculations using the Dock6 protocol. In the force field of MMFF94, the 3D structure of puerarin was established using energy minimization. A crystal/NMR structure of the SIRT1 protein (PDB code: 4ZZH, resolution: 3.1 Å) was obtained from the RCSB Protein Data Bank (http://www.rcsb.org/). Using the crystal ligand, the binding pocket was determined. The DOCK 6.9 program was used for semiflexible docking, and the Grid scoring function was used to analyze the output poses [28, 29].

### Cell counting Kit-8 (CCK8) experiment

The optimal concentration of puerarin was identified using a CCK8 assay kit (Biosharp, China, Cat. No. BS350B). AML12 cells were inoculated in a 96-well plate and treated with different puerarin concentrations (0, 50, 75, 100, 125, 150, 200 μM) for 24 h. Then, 10 μl of CCK-8 solution was added to each well and incubated at 37 °C for 2 h. Finally, a microplate reader (Thermo Scientific, USA, Varioskan Flash) was used to detect the absorbance at 450 nm.

### Determination of iron levels

A tissue iron assay kit (Solarbio, China, BC4355) was used to measure total liver tissue iron concentrations. Liver tissue (0.1 g) was homogenized in an ice bath with 1 ml of extraction solution. The sample was centrifuged at 4000 g and 4 °C for 10 min, and the supernatant was used for subsequent measurements according to the manufacturer’s instructions. Using a microplate reader (Thermo Scientific, USA, Varioskan Flash), the absorbance value was measured at 520 nm.

A ferrous ion assay kit (Solarbio, China, BC5415) was used to measure Fe^2+^ levels in liver tissue. Liver tissue (0.1 g) was homogenized in an ice bath in 1 ml of Reagent I. The sample was centrifuged at 10,000 × g for 10 min at 4 °C, and the supernatant was used for subsequent measurements according to the manufacturer’s instructions. Using a microplate reader (Thermo Scientific, USA, Varioskan Flash), the absorbance value was measured at 593 nm.

### FerroOrange staining

Using a FerroOrange kit (Dojindo, Shanghai, China, Cat. No. F374), intracellular Fe^2+^ levels were detected. AML12 cells were incubated with 1 μM FerroOrange working solution at 37 °C for 30 min, and then the fluorescence intensity was detected using a fluorescence microscope (Andor, Britain, BC43).

### Detection of ROS

The liver paraffin sections were incubated with a 1% dihydroethidium (DHE) solution (Beyotime, China, Cat. No. S0063) for 30 min at 37 °C, and fluorescence was observed by fluorescence microscopy (Olympus, Japan, BX53) at an excitation wavelength of 580 nm.

### Statistical analysis

The data are reported as the mean ± SD, and the experiments were repeated at least three time. The data were analyzed using GraphPad 8.0 software, one-way analysis of variance and t tests, and *P* < 0.05 was considered to indicate a significant difference.

## Results

### Puerarin improves glucose metabolism, liver dysfunction, and hyperlipidemia in MAFLD mice

**Fig. 1:**
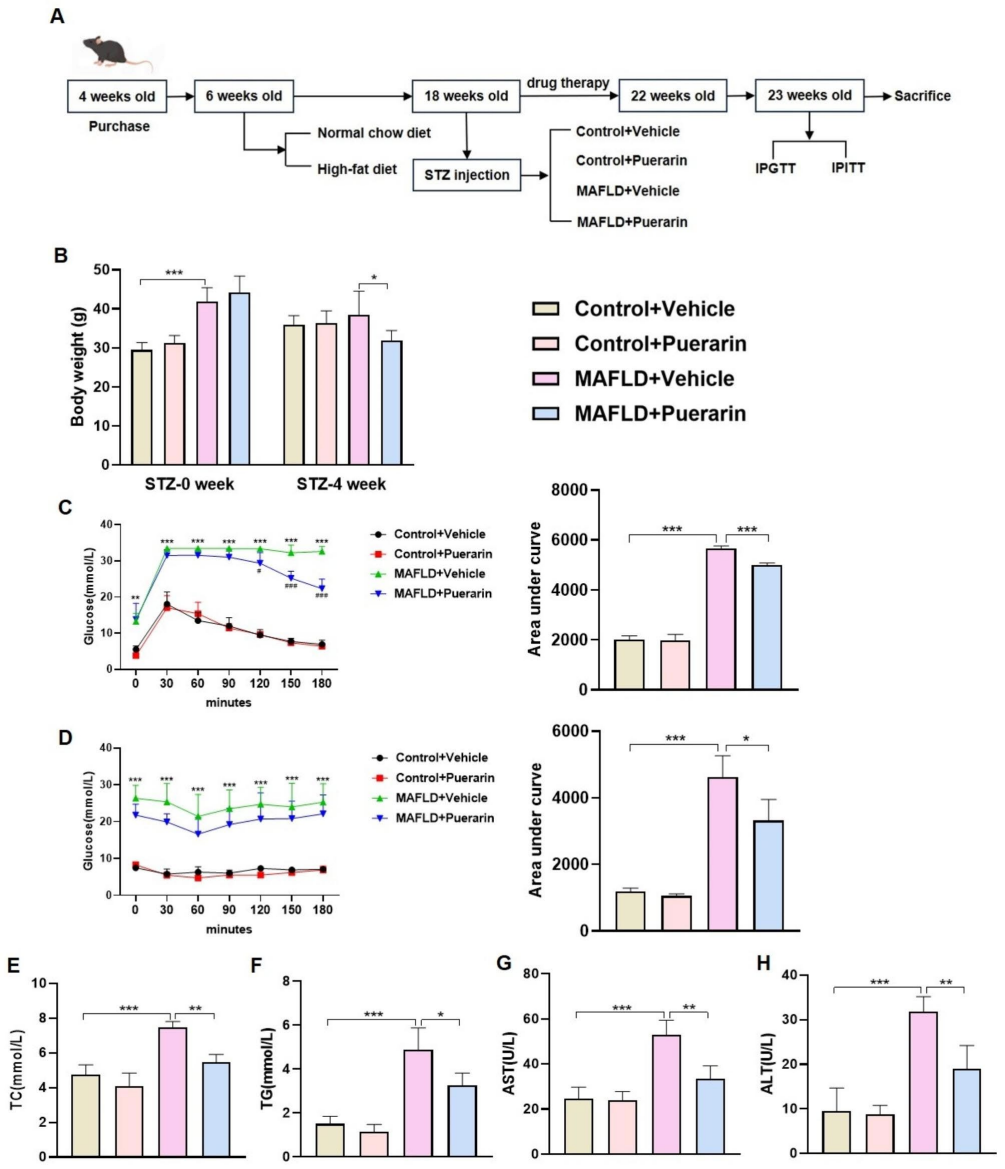
Puerarin improves glucose metabolism, liver dysfunction, and hyperlipidemia in MAFLD mice. (**A**) Flowchart of this investigation. (**B**) Body weights of mice in the Control + Vehicle, Control + Puerarin, MAFLD + Vehicle, and MAFLD + Puerarin groups. (**C**) Intraperitoneal glucose tolerance test (IPGTT) results and area under the curve. (**D**) Intraperitoneal insulin tolerance test (IPITT) results and area under the curve. (**E**-**H**). Measurement of total cholesterol (TC), triglyceride (TG), aspartate aminotransferase (AST), and alanine aminotransferase (ALT) levels in control and MAFLD mice treated with vehicle or puerarin. The data are expressed as the mean ± SD (*n* = 6). *P < 0.05, **P < 0.01, ***P < 0.001 versus Control + Vehicle. ^**#**^*P* < 0.05, ^***##***^*P <* 0.01, ^***###***^*P <* 0.001 versus MAFLD + Vehicle.

In order to confirm the impact of puerarin on MAFLD, A mouse model of MAFLD was created by combined HFD with STZ injection. Subsequently, the mice were treated with either vehicle or puerarin, as depicted in Fig. 1A. Weekly monitoring of body weight revealed a significant increase in HFD-fed mice compared to controls before STZ injection, and the body weights of MAFLD mice decreased following puerarin treatment. However, no statistically significant difference was observed in comparison to the control group (Fig. 1B). The IPGTT and IPITT were used to evaluate the glucose tolerance and insulin sensitivity of MAFLD mice. When compared to the Vehicle group of MAFLD mice, the MAFLD + Puerarin group showed a significant decrease in the area under the curve, indicating that puerarin may improve glucose tolerance (Fig. 1C, D). Moreover, liver enzymes and blood lipid levels in the different groups were analyzed showed that MAFLD mice had significantly higher ALT and AST levels, indicating impaired liver function. Additionally, MAFLD mice exhibited increased TG and TC levels. Figure 1E-H shows that puerarin decreased ALT, AST, TG, and TC levels. These data collectively suggest that puerarin can improve glucose metabolism, liver dysfunction, and hyperlipidemia in MAFLD mice.

### Puerarin ameliorates pathological changes, oxidative stress, and iron overload in MAFLD mice

**Fig. 2:**
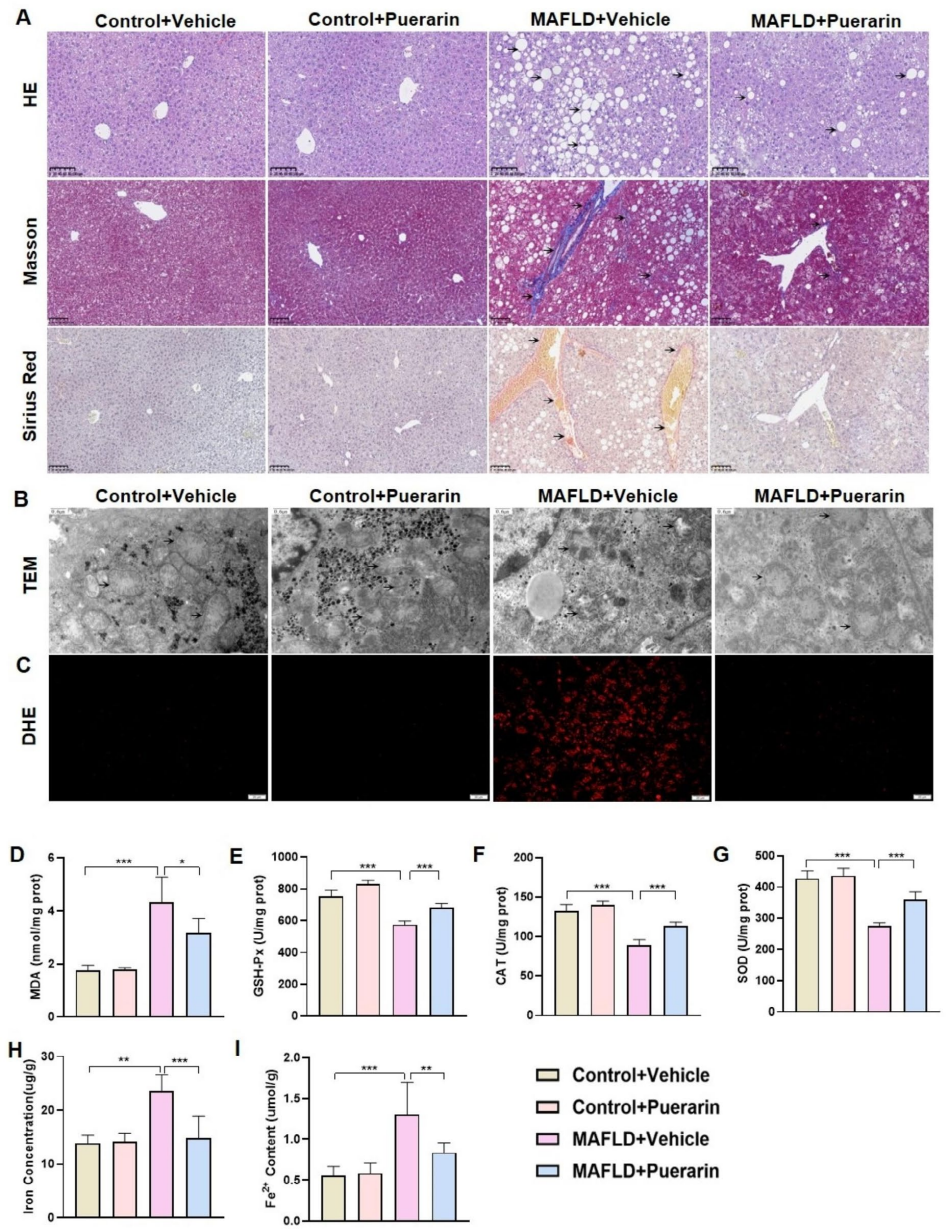
Puerarin ameliorates pathological alterations, oxidative stress, and iron overload in MAFLD mice. (**A**) HE staining, Masson staining, and Sirius red staining in control and MAFLD mice treated with vehicle or puerarin. Scale Bar, 100 μm. (**B**) Assessment of mitochondrial morphology in hepatocytes by TEM. Scale Bar, 0.6 μm. (**C**) Representative images of DHE staining in mouse liver sections. Scale Bar, 20 μm. (**D**-**G**). Measurement of malondialdehyde (MDA), glutathione peroxidase (GSH-Px), catalase (CAT), and superoxide dismutase (SOD) levels in liver tissue in the four groups. (**H**-**I**). Measurement of iron concentrations and Fe^2+^ levels in the livers of mice. The data are expressed as the mean ± SD (*n* = 6). *P < 0.05, **P < 0.01, ***P < 0.001

Furthermore, the impact of puerarin on liver histological structure was examined. HE staining revealed significant vacuolar changes and steatosis in MAFLD mice compared to control mice. Furthermore, Masson staining and Sirius red staining showed significant liver fibrosis in MAFLD mice. However, puerarin treatment could improve the disordered pathological structure and liver fibrosis. Although the liver macrovescicular steatosis was reduced in MAFLD + Puerarin group, it was still present. (Fig. 2A). Ferroptosis has been shown to contribute to the development and progression of MAFLD in recent studies. TEM revealed typical mitochondrial morphological features of ferroptosis in hepatocytes in MAFLD mice, including reduced mitochondrial size, increased membrane density, and decreased cristae. However, puerarin treatment attenuated mitochondrial damage and restored mitochondrial morphology (Fig. 2B). Iron overload and lipid peroxidation are essential for the progression of ferroptosis. Intracellular ROS levels were detected by DHE staining and were significantly increased in MAFLD mouse hepatocytes compared to control mouse hepatocytes, while puerarin treatment significantly reduced ROS levels (Fig. 2C). Furthermore, there was a notable rise in MDA concentrations, while GSH-Px, CAT, and T-SOD levels experienced a significant decline in MAFLD mice, indicating an elevation in liver oxidative stress (Fig. 2D - G). Interestingly, as a result of puerarin treatment, MDA levels decreased while GSH-Px, CAT, and SOD levels increased. In addition, there was a notable rise in iron concentrations and Fe^2+^ levels in MAFLD mice when compared to control mice, and puerarin inhibited iron overload and reduced iron concentrations and Fe^2+^ levels in MAFLD mice (Fig. 2H, I). Collectively, puerarin can ameliorate pathological changes, reduce oxidative stress, and mitigate hepatocyte iron overload in MAFLD mice.

### Puerarin inhibits hepatic ferroptosis and activates the SIRT1/Nrf2 signaling pathway in MAFLD mice

**Fig. 3:**
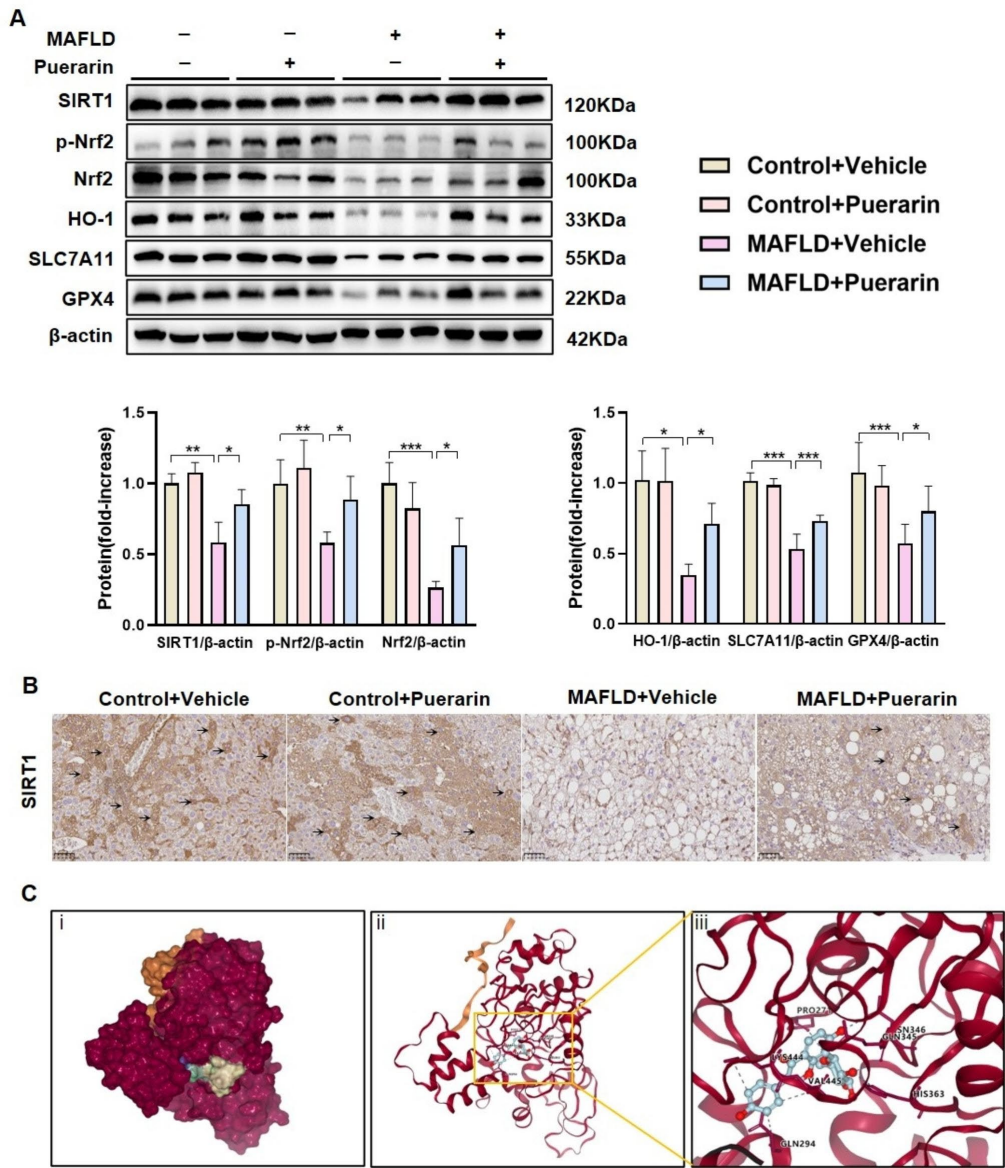
Puerarin inhibits hepatic ferroptosis and activates the SIRT1/Nrf2 signaling pathway in MAFLD mice. (**A**) The protein levels of SIRT1, p-Nrf2, Nrf2, HO-1, SLC7A11, and GPX4 were determined by Western blotting. (**B**) Representative images of immunohistochemical staining showing the expression of SIRT1 in liver sections. (**C**) Molecular docking model of SIRT1 and Puerarin (i. Spherical diagram, ii. 3D structures of the binding interface, iii. Puerarin was predicted to interact with SIRT1 via the amino acids PRO271, GLN345, ASN346, HIS363, GLN294, LYS444, and VAL445 via hydrophobic interactions or hydrogen bonds. The data are expressed as the mean ± SD (*n* = 5). *P < 0.05, **P < 0.01, ***P < 0.001

Furthermore, key proteins involved in inhibiting ferroptosis were examined, such as solute carrier family-7 member-11 (SLC7A11) and Glutathione peroxidase 4 (GPX4). Compared to those in control mice, SLC7A11 and GPX4 protein levels were considerably lower in MAFLD mice. However, puerarin treatment increased the protein levels of SLC7A11 and GPX4, demonstrating that puerarin could inhibit ferroptosis (Fig. 3A). We then examined whether puerarin could regulate the expression of Nrf2, which is a ferroptosis inhibitor, and its upstream and downstream targets. According to Western blot analysis, the MAFLD group’s SIRT1 levels were significantly lower than the controls’, as well as the levels of p-Nrf2, Nrf2, and HO-1. MAFLD mice treated with puerarin, however, showed significant upregulation of SIRT1, p-Nrf2, Nrf2, and HO-1 expression (Fig. 3A). Consistent with the Western blot results, the immunohistochemical results confirmed the reduced expression of SIRT1 in MAFLD mice compared to control mice, while puerarin treatment increased SIRT1 expression (Fig. 3B). The binding mode of puerarin to SIRT1 and the specific amino acid residues involved in this interaction were investigated using molecular docking analysis. The grid score was calculated based on electrostatic interactions and van der Waals interactions between puerarin and the binding amino acid of SIRT1 and was − 59.488323 kcal·mol^− 1^, suggesting stable binding between puerarin and SIRT1. We found that puerarin could bind within the cap binding site in SIRT1 through hydrophobic interactions or hydrogen bonds with amino acids PRO271, GLN345, ASN346, HIS363, GLN294, LYS444, and VAL445 (Fig. 3C). These results indicated that puerarin could inhibit hepatic ferroptosis and activate the SIRT1/Nrf2 signaling pathway in MAFLD mice.

### Puerarin increases Nrf2 nuclear translocation and reduces liver inflammation in MAFLD mice

**Fig. 4:**
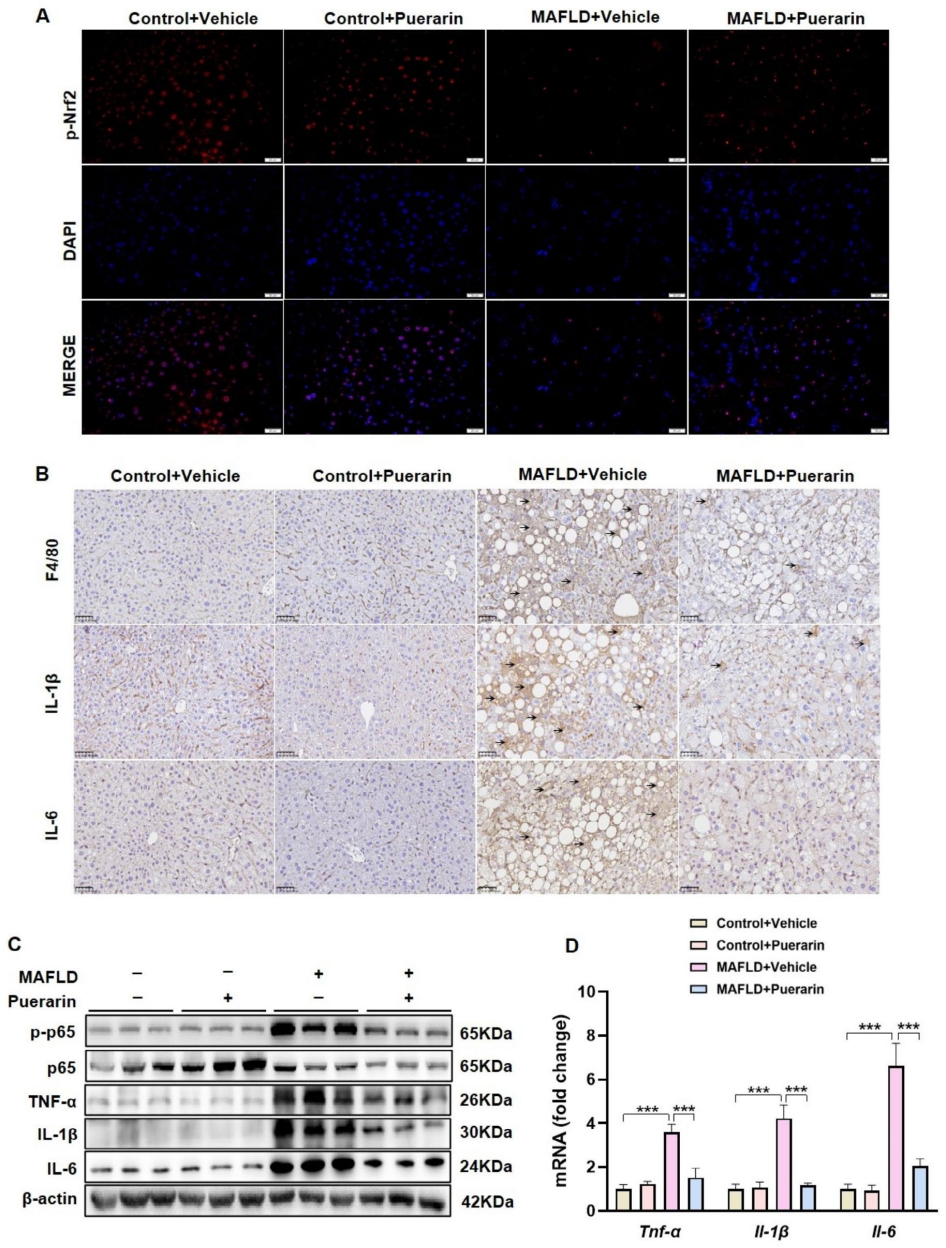
Puerarin increases Nrf2 nuclear translocation and reduces liver inflammation in MAFLD mice. (**A**) Representative images of immunofluorescence staining of p-Nrf2. Scale Bar, 20 μm. (**B**) F4/80, IL-1β, and IL-6 were determined by immunohistochemical staining. Scale Bar, 50 μm. (**C**) The protein levels of p-p65/p65, TNF-α, IL-1β, and IL-6 were determined by Western blotting. (**D**) Hepatic mRNA levels of *Tnf-α*, *Il-1β*, and *Il-6* were determined by RT‒qPCR. The data are expressed as the mean ± SD (*n* = 4). *P < 0.05, **P < 0.01, ***P < 0.001

According to immunofluorescence staining, MAFLD mice had reduced Nrf2 nuclear translocation compared with control mice, and puerarin treatment enhanced Nrf2 nuclear translocation (Fig. 4A). Moreover, we investigated the impact of puerarin on inflammation in MAFLD. In MAFLD mice, the immunohistochemical findings indicated that puerarin suppressed the infiltration of F4/80-positive macrophages and reduced the levels of IL-1β and IL-6 expression. (Fig. 4B). The Western blot results showed that puerarin reduced the level of phosphorylated p65 and decreased the protein levels of TNF-α, IL-1β, and IL-6 in MAFLD mice, indicating that puerarin could inhibit the NF-κB p65 pathway in MAFLD mice (Fig. 4 C, S1A-B). The RT‒qPCR results revealed a substantial increase in the mRNA levels of *Tnf-α*, *Il-1β*, and *Il-6* in MAFLD mice, and puerarin reduced the transcript levels of these proinflammatory cytokines (Fig. 4D). These results suggest that puerarin promotes Nrf2 nuclear translocation and reduces liver inflammation in MAFLD mice.

### Puerarin inhibits ferroptosis, activates the SIRT1/Nrf2 signaling pathway, and reduces inflammation in PA-induced AML12 cells in vitro

**Fig. 5:**
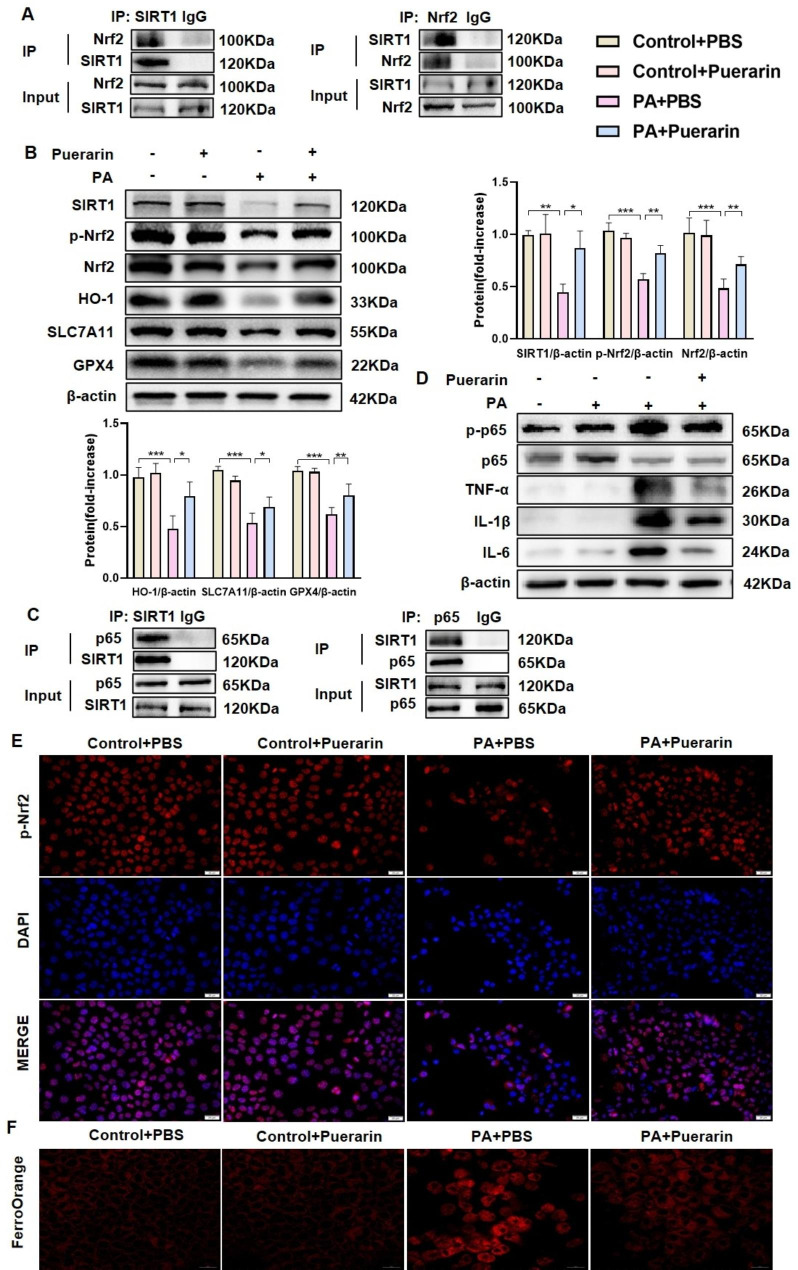
Puerarin inhibits ferroptosis, activates the SIRT1/Nrf2 signaling pathway, and reduces inflammation in PA-induced AML12 cells in vitro. (**A**) Coimmunoprecipitation was performed to assess the interaction between SIRT1 and Nrf2 in AML12 cells. (**B**) Protein levels of SIRT1, p-Nrf2, Nrf2, HO-1, SLC7A11, and GPX4 in AML12 cells in the Control + PBS, Control + Puerarin, PA + PBS, and PA + Puerarin groups. (**C**) Coimmunoprecipitation was performed to assess the interaction between SIRT1 and p65 in AML12 cells. (**D**) Protein levels of p-p65/p65, TNF-α, IL-1β, and IL-6 in the four groups. (**E**) Representative images of immunofluorescence staining of p-Nrf2 in AML12 cells. Scale Bar, 20 μm. (**F**) Representative images of Ferrorange staining in the four groups. Scale Bar, 20 μm. The data are expressed as the mean ± SD (*n* = 4). *P < 0.05, **P < 0.01, ***P < 0.001

In order to investigate the underlying mechanism of the protective effect of puerarin, a coimmunoprecipitation assay was performed to comfirm the interaction between SIRT1 and Nrf2 in AML12 cells (Fig. 5A). In vitro, the cell model of MAFLD was created by inducing AML12 cells with PA. The optimal concentration of puerarin was determined to be 75 μM based on the CCK8 assay, and this concentration did not affect cell viability (Figure S2A). Subsequently, this study examined the signaling pathways associated with SIRT1/Nrf2, inflammation, and ferroptosis. Western blot analysis revealed that SIRT1, p-Nrf2, Nrf2, and HO-1 protein levels were decreased in AML12 cells in response to PA stimulation compared with those in the control group. However, puerarin treatment significantly prevented the PA-induced reduction in SIRT1, p-Nrf2, Nrf2, and HO-1 and upregulated SLC7A11 and GPX4 protein expression (Fig. 5B). Consistently, the coimmunoprecipitation results confirmed the interaction between SIRT1 and p65 (Fig. 5C). Furthermore, puerarin downregulated the NF-κB/p65 pathway, leading to decreased protein expression of the proinflammatory factors TNF-α, IL-1β and IL-6 in PA-induced AML12 cells (Fig. 5D, Figure S3A-B). The RT‒qPCR results showed that puerarin decreased the expression of the proinflammatory cytokines *Tnf-α, Il-1β*, and *Il-6* in PA-induced AML12 cells (Figure S3C). In PA-induced AML12 cells, puerarin promoted the nuclear translocation of Nrf2, according to immunofluorescence analysis (Fig. 5E). Additionally, FerroOrange staining indicated that puerarin reduced intracellular Fe^2+^ levels in AML12 cells induced by PA (Fig. 5F). Based on these results, puerarin inhibits ferroptosis, activates SIRT1/Nrf2 signaling pathways, and reduces inflammation in PA-induced AML12 cells.

### The protective effect of puerarin on PA-induced AML12 cells is reduced by inhibiting the SIRT1/Nrf2 signaling pathway

**Fig. 6:**
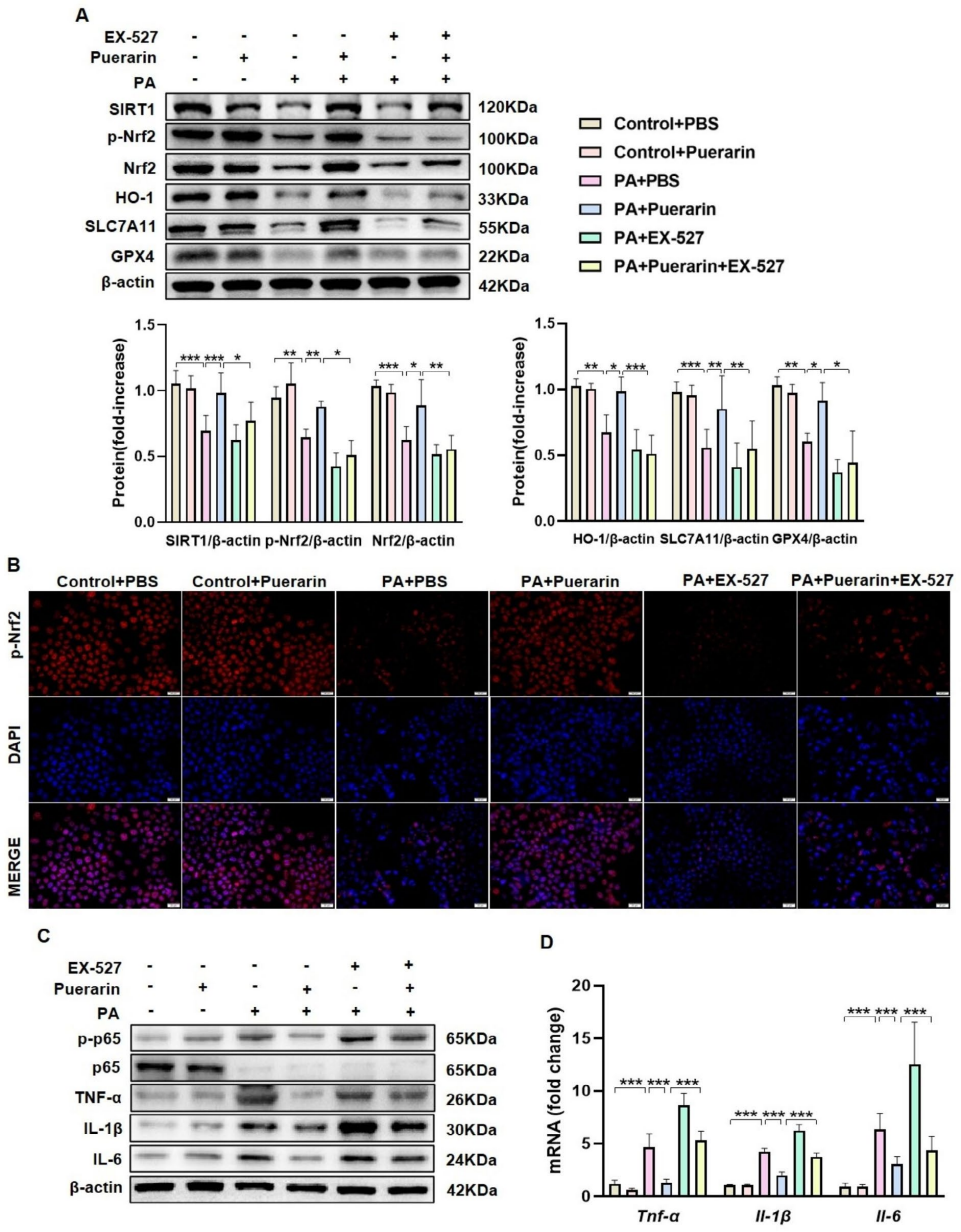
SIRT1 inhibition reduced the protective effect of puerarin on PA-induced AML12 cells. (**A**) Protein levels of SIRT1, p-Nrf2, Nrf2, HO-1, SLC7A11, and GPX4 in AML12 cells in the Control + PBS, Control + Puerarin, PA + PBS, PA + Puerarin, PA + EX-527 (10 μM), and PA + Puerarin + EX-527 (10 μM) groups. (**B**) Representative images of immunofluorescence staining of p-Nrf2 in AML12 cells. Scale Bar, 20 μm. (**C**) The protein levels of p-p65/p65, TNF-α, IL-1β, and IL-6 in AML12 cells in the six groups. (**D**) The mRNA levels of *Tnf-α*, *Il-1β*, and *Il-6* in AML12 cells in the six groups. The data are expressed as the mean ± SD (*n* = 4). *P < 0.05, **P < 0.01, ***P < 0.001

**Fig. 7:**
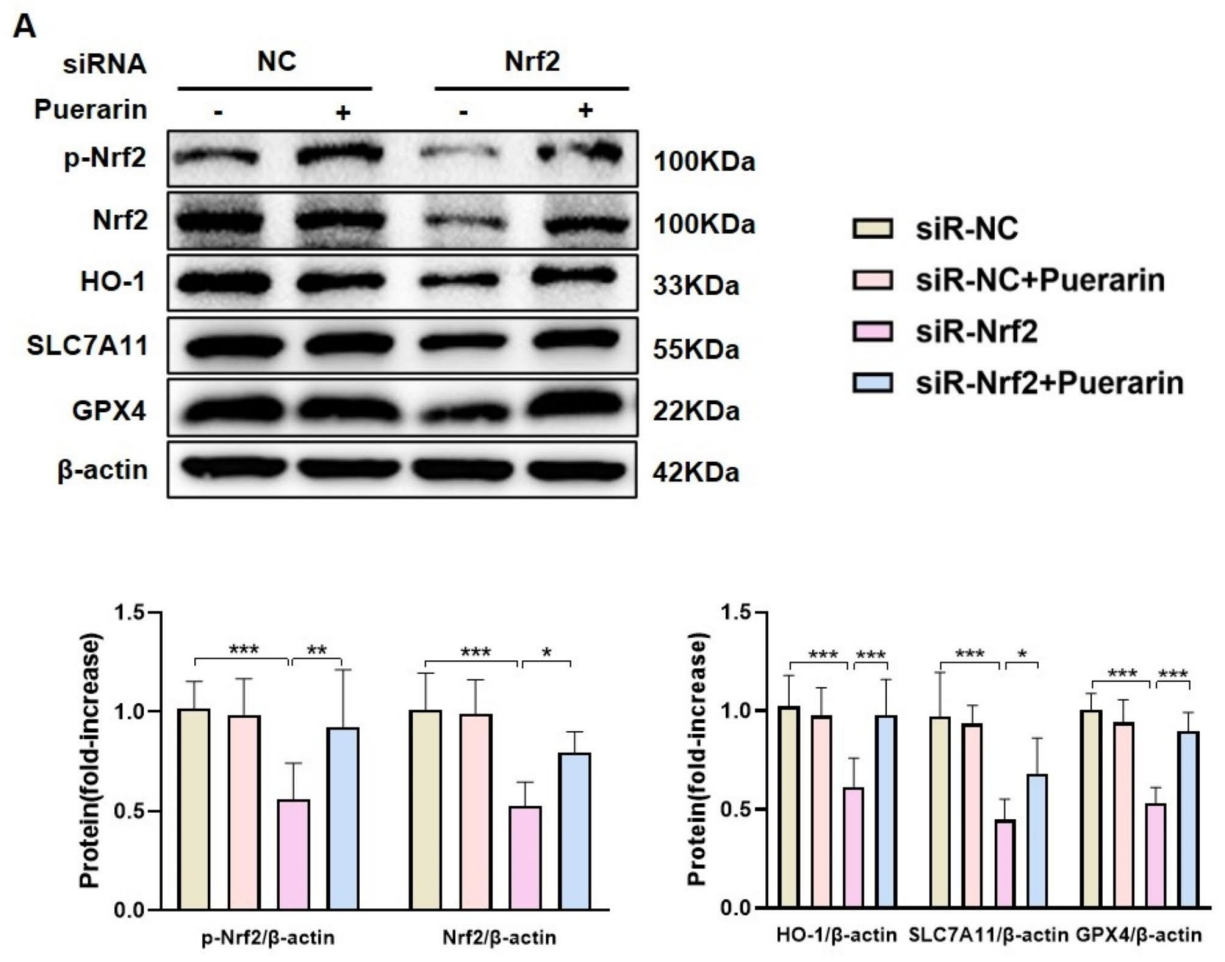
Nrf2 knockdown reduces puerarin-mediated inhibition of ferroptosis in PA-induced AML12 cells. **A**. Protein levels of p-Nrf2, Nrf2, HO-1, SLC7A11, and GPX4 in AML12 cells in the siRNA-NC, siRNA-NC + puerarin, siRNA-Nrf2, and siRNA-Nrf2 + puerarin groups. The data are expressed as the mean ± SD (*n* = 4). *P < 0.05, **P < 0.01, ***P < 0.001

To assess the significance of SIRT1, AML12 cells underwent pretreatment with EX-527, a SIRT1 inhibitor. EX-527 partially eliminated puerarin-mediated increases in SIRT1, p-Nrf2, Nrf2, and HO-1 protein levels and Nrf2 nuclear translocation in PA-induced AML12 cells (Fig. 6A, B). Additionally, Furthermore, the Western blot and RT‒qPCR findings indicated that EX-527 partially counteracted the inhibitory impact of puerarin on proinflammatory markers in PA-induced AML12 cells (Fig. 6 C-D, S4A-B), and SLC7A11 and GPX4 protein levels were not upregulated in AML12 cells (Fig. 6A). To investigate the role of Nrf2 in these processes, siRNA was used to knockdown Nrf2 in AML12 cells (Figure S5A). After Nrf2 was knocked down, puerarin failed to enhance the protein expressions of HO-1, SLC7A11, and GPX4 in AML12 cells induced by PA (Fig. 7A). These findings demonstrate that the protective effect of puerarin on MAFLD was dependent on SIRT1/Nrf2 signaling pathway.

## Discussion

Puerarin has been shown to improve NAFLD by reducing hepatic lipid deposition and oxidative stress [30], however, there are no explicit reports elucidating the role of puerarin on MAFLD in regulating ferroptosis and inflammation. This study established a MAFLD mouse model with hyperglycemia and hyperlipidemia to investigate the effect of puerarin treatment on MAFLD, and specifically concentrated on identifying therapeutic targets and understanding the underlying mechanisms.

Ferroptosis is a form of programmed cell death that is iron dependent. Unlike necrosis, apoptosis, and autophagy, ferroptosis is primarily caused by iron overload [31]. The Fenton reaction converts Fe^2+^ to Fe^3+^ and generates ROS, while excess iron promotes the Fenton reaction, leading to oxidative stress and accelerating lipid peroxidation by activating lipoxygenase. This results in excessive deposition of lipid peroxides, causing cell damage and death [32]. GPX4, the central inhibitor of ferroptosis, is an antioxidant enzyme that plays a crucial role in inhibiting lipid peroxidation. Glutathione (GSH) is produced by the activation of SLC7A11, a cystine-glutamate counter transporter protein, and is an important cofactor in maintaining normal GPX4 function [33]. Inhibiting SLC7A11 leads to GSH depletion, further inactivating GPX4. In this study, TEM revealed distinct morphological characteristics in hepatocytes, distinguishing typical ferroptosis from necrosis and apoptosis. This study also revealed for the first time that puerarin treatment restored mitochondrial morphology, reduced iron overload, and decreased lipid peroxide accumulation, thus inhibiting ferroptosis.

Redox imbalance generates large amounts of ROS, which specifically target lipids and cause lipid peroxide deposition. The restoration of redox homeostasis can effectively reduce lipid peroxidation and inhibit ferroptosis. The transcription factor Nrf2 plays a key role in regulating the intracellular antioxidant response and can translocate into the nucleus to initiate the transcription of antioxidant response elements (AREs). Downstream proteins of the Nrf2-ARE pathway include HO-1, superoxide dismutase, glutamate cysteine ligase, glutathione peroxidase, and glutathione S-transferase [34]. SIRT1, which is an important energy receptor in various metabolic tissues, exerts important regulatory effects on energy metabolism, oxidative stress, and inflammation. Activation of SIRT1 can reduce the expression of proinflammatory cytokines and the infiltration of macrophages in the liver and adipose tissue by deacetylating and downregulating the transcriptional activity of NF-κB [35]. Moreover, SIRT1 can deacetylate Nrf2, enhancing its activation and translocation into the nucleus to bind ARE promoter regions and increase the transcription of antioxidant enzymes [36]. Nrf2 regulates the transcription of three major classes of ferroptosis-related genes to inhibit ferroptosis: iron metabolism-related genes (such as ferritin heavy chain 1, HO-1, metallothionein 1 G), genes involved in the synthesis, metabolism, and release of GSH (such as SLC7A11, glutathione synthetase, and ATP binding cassette subfamily C member 1), and genes involved in detoxification or antioxidant responses (such as NAD(P)H: quinone oxidoreductase 1, sestrin 2, and glutathione S-transferases pi 1) [37]. Among them, the HO-1 protein participates in iron metabolism and is an essential antioxidant enzyme.

This study demonstrated that puerarin could activate the SIRT1/Nrf2 pathway in MAFLD mice and PA-induced AML12 cells, leading to increased protein expression of HO-1, SLC7A11, and GPX4 and effectively suppressing ferroptosis. Significantly, the beneficial impact of puerarin on PA-induced AML12 cells vanished upon utilization of the SIRT1 inhibitor (EX-527), and the increase in HO-1, SLC7A11, and GPX4 protein levels induced by puerarin was no longer observed when Nrf2 was knocked down. The findings indicate that the SIRT1/Nrf2 signaling pathway is crucial in regulating puerarin-mediated inhibition of ferroptosis and inflammation.

### Study strengths and limitations

This study has several strengths. First, ferroptosis was found to be involved in the development of MAFLD, and there have been limited investigations on the relationship between SIRT1, MAFLD and ferroptosis. Secondly. This study further explored that puerarin improved MAFLD by modulating SIRT1/Nrf2 signaling pathway to enhance the anti-lipid peroxidation ability and thus inhibit ferroptosis. However, there are several limitations of this study. In this research, the SIRT1 inhibitor EX-527 was exclusively utilized for in vitro experiments to confirm if SIRT1 has a crucial function in the puerarin-induced suppression of ferroptosis, and in-depth mechanistic investigations in vivo are needed. Second, this study focused on the regulatory function of lipid peroxidation in ferroptosis inhibition by puerarin, and although it was observed that puerarin was able to reduce iron content, no in-depth mechanistic investigation was performed. Finally, puerarin was able to decrease body weight and improve glucose and insulin sensitivity in MAFLD mice, and whether the inhibitory effect of puerarin on ferroptosis was secondary to metabolic remission deserves further investigation.

## Conclusion

**Fig. 8:**
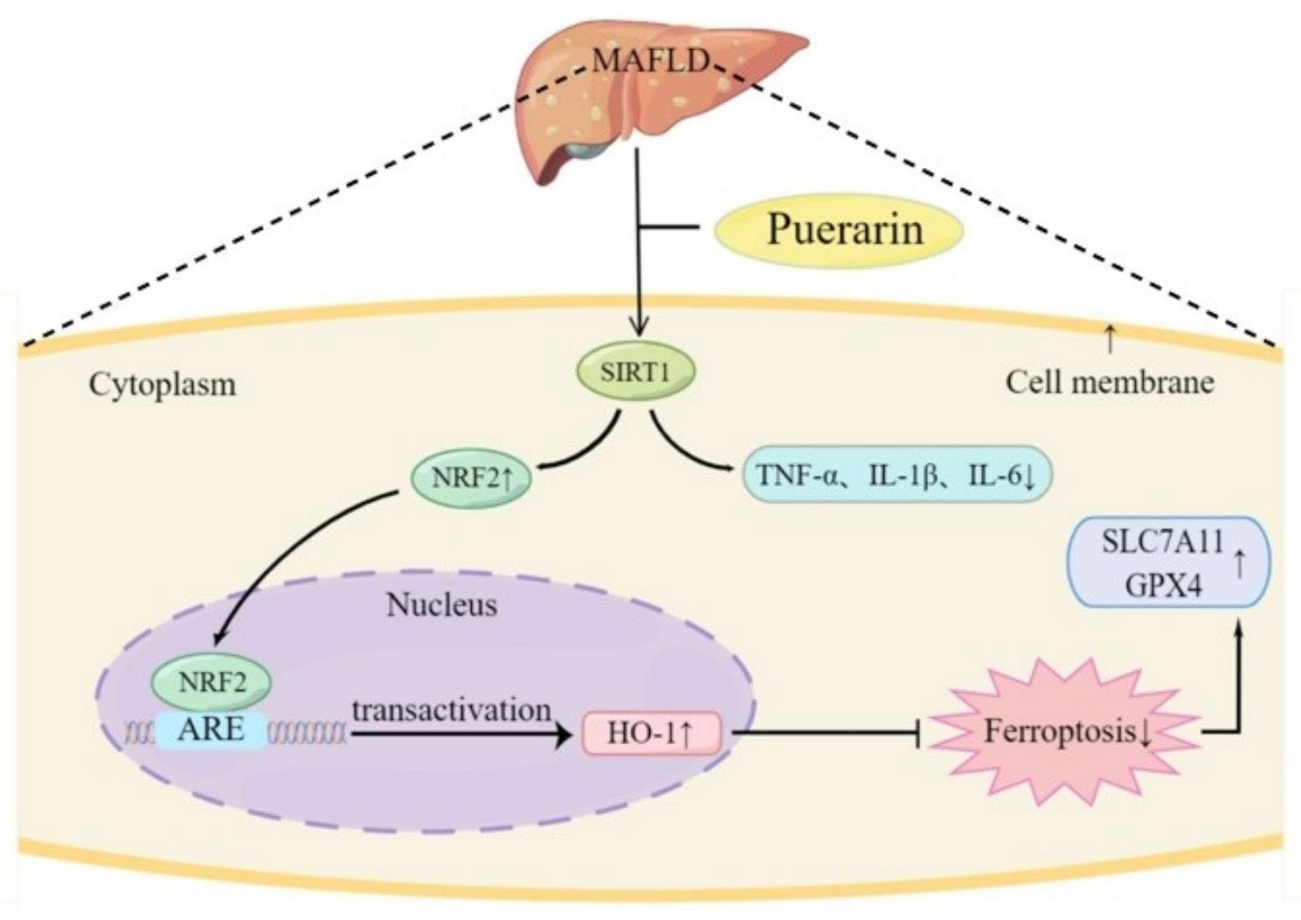
Proposed antiferroptotic and anti-inflammatory mechanism of puerarin. Puerarin ameliorates metabolic dysfunction-associated fatty liver disorder by suppressing ferroptosis and inflammation via the SIRT1/Nrf2 signaling pathway

The findings of this research indicate that ferroptosis shows potential as a viable approach for intervention in MAFLD, and that puerarin can improve MAFLD via the SIRT1/Nrf2 signaling pathway, thereby suppressing ferroptosis and inflammation (Fig. 8). Puerarin is a class of flavonoids with various pharmacological properties. Puerarin and its derivatives can be added to daily diets for the prevention of chronic metabolic diseases, and can also be extended to the primary prevention of MAFLD in clinical practice.

### Electronic supplementary material

Below is the link to the electronic supplementary material.

Supplementary Material 1

## Data Availability

The data supporting the outcomes of this investigation are accessible from the corresponding author upon request.

## Abbreviations

4-HNE: 4-hydroxynonenal
ALT: Alanine aminotransferase
AST: Aspartate aminotransferase
CAT: Catalase
DHE: Dihydroethidium
GPX4: Glutathione peroxidase 4
GSH-Px: Glutathione peroxidase
HE: Hematoxylin-eosin
HFD: High-fat diet
HO-1: Heme oxygenase-1
IPGTT: Intraperitoneal glucose tolerance test
IPITT: Intraperitoneal insulin tolerance test
MAFLD: Metabolic dysfunction associated fatty liver disease
MDA: Malondialdehyde
NAFLD: Non-alcoholic fatty liver disease
NF-κB: Nuclear factor-κB
Nrf2: Nuclear factor erythroid 2-related factor 2
PA: Palmitic acid
PUFA-PL: Phospholipid of polyunsaturated fatty acids
PUFAs: Polyunsaturated fatty acids
ROS: Reactive oxygen species
SLC7A11: Solute carrier family-7 member-11
SOD: Superoxide dismutase
STZ: Streptozotocin
T2DM: Type 2 diabetes mellitus
TC: Total cholesterol
TEM: Transmission electron microscopy
TG: Triglyceride
