# Supplementary material for: Puerarin ameliorates metabolic dysfunction-associated fatty liver disease by inhibiting ferroptosis and inflammation
Source: Lipids Health Dis. 2023 Nov 24;22:202. doi: 10.1186/s12944-023-01969-y (PMC10668385; doi:10.1186/s12944-023-01969-y)
Supplement: Supplementary file 1 — Supplementary Material 1 [file 12944_2023_1969_MOESM1_ESM.docx]

Table S1. Compositions of experimental diets

|  | High-fat diet | Normal chow diet |
| --- | --- | --- |
| Energy (kcal/kg diet) | 5240 | 3850 |
| Protein (kcal %) | 20 | 20 |
| Carbohydrate (kcal %) | 20 | 70 |
| Fat (kcal %) | 60 | 10 |
| Ingredient gm % (kcal %) |  |  |
| Casein | 200 (800) | 200 (800) |
| L-Cystine | 3 (12) | 3 (12) |
| Corn Starch | 0 (0) | 506.2 (2024.8) |
| Maltodextrin | 125 (500) | 125 (500) |
| Sucrose | 68.8 (275) | 68.8 (275) |
| Cellulose, BW200 | 50 (0) | 50 (0) |
| Soybean Oil | 25 (225) | 25 (225) |
| Lard | 245 (2205) | 20 (180) |
| Mineral Mix S10026 | 10 (0) | 10 (0) |
| DiCalcium Phosphate | 13 (0) | 13 (0) |
| Calcium Carbonate | 5.5 (0) | 5.5 (0) |
| Potassium Citrate, 1 H20 | 16.5 (0) | 16.5 (0) |
| Vitamin Mix V10001 | 10 (40) | 10 (40) |
| Choline Bitartrate | 2 (0) | 2 (0) |
| FD&C Yellow Dye **#** 5 | 0 (0) | 0.4 (0) |
| FD&C Red Dye **#** 40 | 0 (0) | 0 (0) |
| FD&C Blue Dye **#** 1 | 0.05 (0) | 0.01 (0) |
| Total | 773.85 (4057) | 1055.05 (4057) |


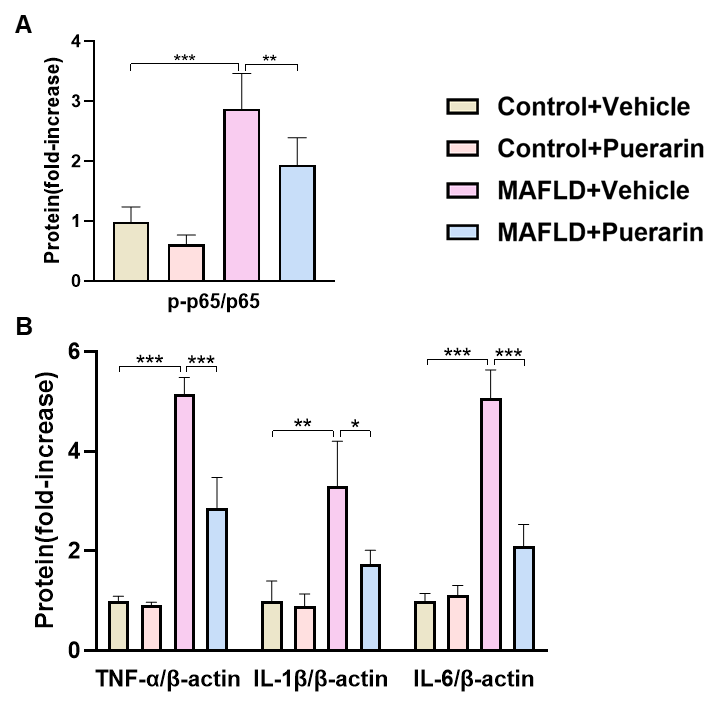


Supplementary Figure 1. A-B. Relative proteins between Control+Vehicle, Control+Puerarin, T2DM+Vehicle, T2DM+Puerarin groups. Densitometric analyses of p-p65/p65, TNF-α, IL-1β, IL-6. Data are expressed as mean ± SD (*n* = 4). **P* < 0.05, ***P* < 0.01, ****P* < 0.001.


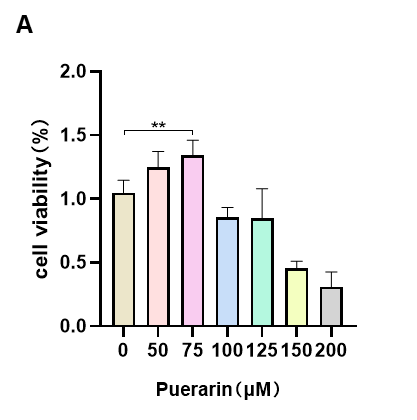


Supplementary Figure 2. A. The cell counting kit 8 (CCK8) assay. The concentration of puerarin (50, 75, 100, 125, 150, 200 μmmol/L) when it was co-incubated with AML12 cells. Data are expressed as mean ± SD (*n* = 3).**P*< 0.05*, **P* < 0.01*, ***P* < 0.001.


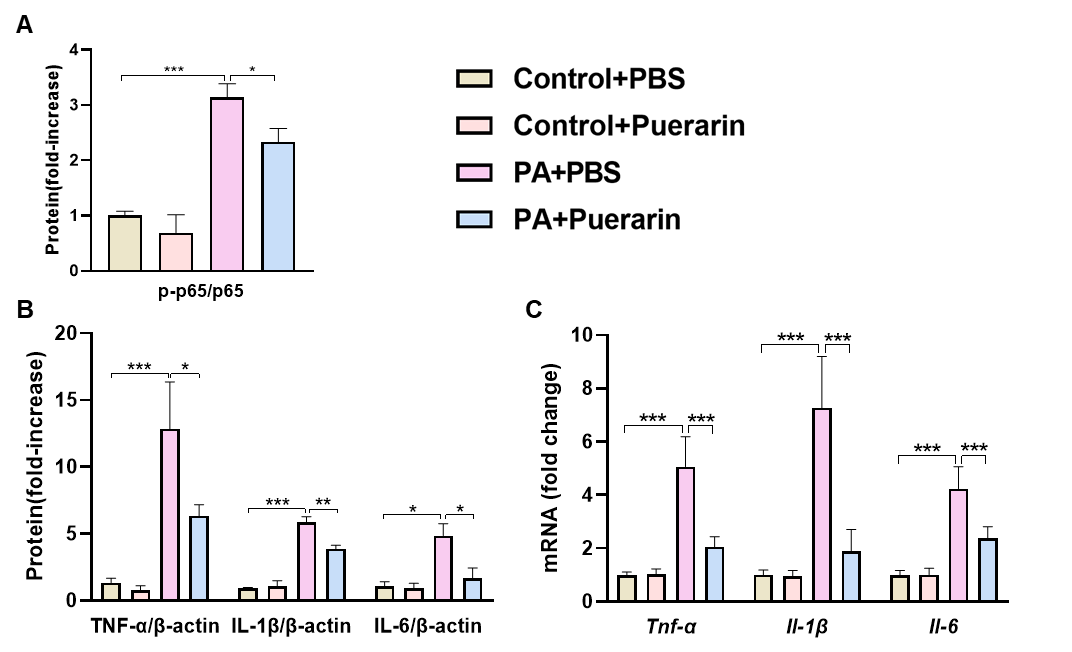


Supplementary Figure 3. A-B. Relative proteins between Control+PBS, Control+Puerarin, PA+PBS, PA+Puerarin groups. Densitometric analyses of p-p65/p65, TNF-α, IL-1β, IL-6. C. AML12 cells mRNA levels of *Tnf-α*, *Il-1β*, and *Il-6* were detected by RT-qPCR. Data are expressed as mean ± SD (*n* = 4). **P* < *0.05*, ***P* < *0.01*, ****P* < *0.001*.


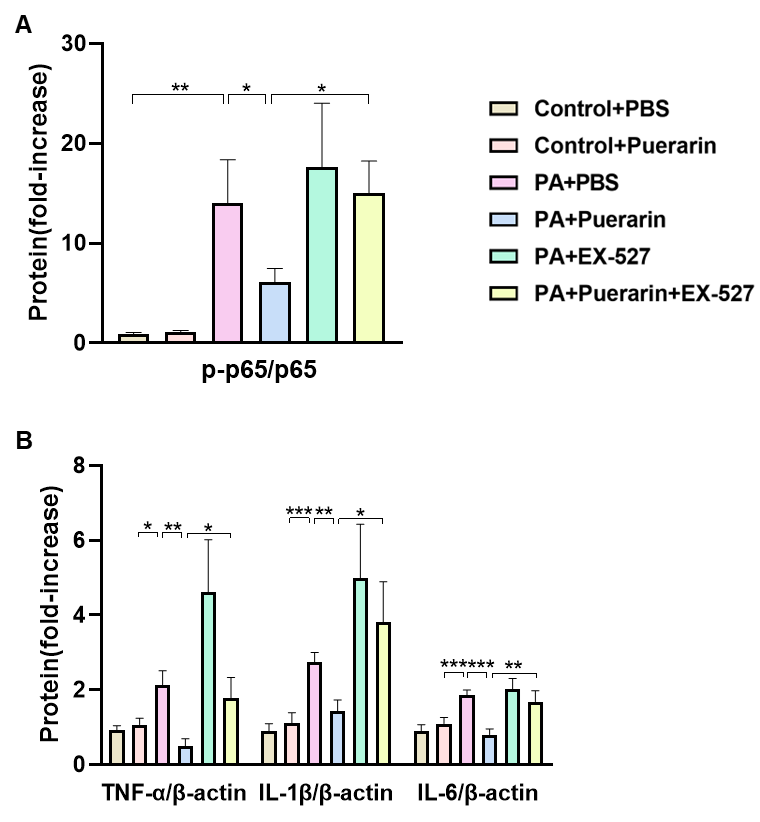
Supplementary Figure 4. A-B. Relative proteins between Control+PBS, Control+Puerarin, PA+PBS, PA+Puerarin, PA+EX-527, PA+Puerarin+EX-527 groups. Densitometric analyses of p-p65/p65, TNF-α, IL-1β, IL-6. Data are expressed as mean ± SD (*n* = 4). **P* < *0.05*, ***P* < *0.01*, ****P* < *0.001*.


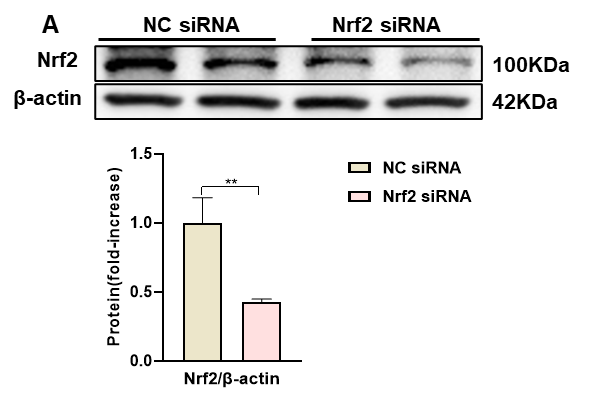


Supplementary Figure 5. A. Nrf2 knockdown verification detected by western blotting. Data are expressed as mean ± SD (*n* = 3).**P*< 0.05*, **P* < 0.01*, ***P* < 0.001.
